# Expert Revision of Key Elements for Clinical-Grade Production and Qualification of Perinatal Derivatives

**DOI:** 10.1093/stcltm/szad068

**Published:** 2023-12-10

**Authors:** Roberto Gramignoli, Nicola Hofmann, Marta Agudo-Barriuso, Mariastefania Antica, Ana I Flores, Lenart Girandon, Halima Kerdjoudj, Ruta Navakauskiene, Jessica Schiavi, Hanne Scholz, Volodymyr Shablii, Xavier Lafarge, Francisco J Nicolás, Florelle Gindraux

**Affiliations:** Department of Laboratory Medicine, Division of Pathology, Karolinska Institutet, Stockholm, Sweden; Department of Pathology and Cancer Diagnostics, Karolinska University Hospital, Stockholm, Sweden; German Society for Tissue Transplantation (DGFG) gGmbH, Hannover, Germany; Experimental Ophthalmology Group, University of Murcia and Instituto Murciano de Investigación Biosanitaria (IMIB), Campus Ciencias de la Salud, Murcia, Spain; Ruder Boskovic Institute, Division of Molecular Biology, Zagreb, Croatia; Regenerative Medicine Group, Research Institute Hospital 12 de Octubre (imas12), Madrid, Spain; Educell Ltd., R&D Department, Trzin, Slowenia; University of Reims Champagne Ardenne, EA 4691 BIOS, Reims, France; Department of Molecular Cell Biology, Institute of Biochemistry, Life Sciences Center, Vilnius, Lithuania; Department of Bioprocesses Biomolecules, University of Lorraine, CNRS, LRGP, Nancy, France; Department of Transplant Medicine, Department of Cellular Therapy, University of Oslo, Oslo, Norway; Laboratory of Biosynthesis of Nucleic Acids, Institute of Molecular Biology and Genetics, Department of Functional Genomics, National Academy of Science, Kyiv, Ukraine; Placenta Stem Cell Laboratory, Cryobank, Institute of Cell Therapy, Kyiv, Ukraine; Etablissement Français du Sang Nouvelle-Aquitaine, Laboratoire d’ingénierie tissulaire et cellulaire, Bordeaux, France; INSERM U1211 « Maladies Rares: Génétique et Métabolisme », Université de Bordeaux, France; Lab. Regeneración, Oncología Molecular y TGFß. IMIB-Pascual Parrilla, El Palmar, Murcia, Spain; CHU Besançon, Service de Chirurgie Maxillo-Faciale, Stomatologie et Odontologie Hospitalière, F-25000 Besançon, France; Université de Franche-Comté, LNIT (Laboratoire de Nanomédecine, Imagerie, Thérapeutique EA 4662), F-25000 Besançon, France

**Keywords:** perinatal derivatives, placenta, European Regulation, cell therapy, ATMP, advanced medical device

## Abstract

Perinatal derivatives have been proposed as adjunct therapeutic strategies or innovative treatments. Undoubtedly, perinatal derivatives can offer the opportunity and source material to isolate multipotent stem cells, but both maternal- and fetal-derived tissues can be processed and transformed into engineered tissues or advanced biomedical devices, whose potential remains to be fully elucidated. Promising preclinical and clinical results collected so far clearly foresee an escalation of such novel treatments. Market forecasts predict exponential growth in such advanced medicinal products during the next decade, with a pragmatic innovation for medicine into a more advanced biomedical version, enlarging the portfolio for treating a wide range of congenital and acute conditions. However, all these promising and fascinating therapeutic possibilities cannot gain a solid and recognized role in established medical practice without rigid and harmonized manufacturing strategies. The implementation of strategies according to guidelines and directives compiled by Regulatory Agencies, in conformity to (European) Pharmacopoeia and for Good Manufacturing Practice -conforming production of such products, represent critical steps required to translate perinatal technologies into effective therapeutic approaches. During the past 5 years, a panel of European experts and developers, gathered under the umbrella of the COST Sprint Action, supported by the European Cooperation in Science and Technology action, had the opportunity to revise and summarize experience and recommendations for a fruitful and proficient generation of perinatal biomedical products. In order to facilitate the creation and potential commercialization of perinatal bioengineered and advanced pharmaceutical products and technologies, such a collection of data and recommendations is described and discussed here.

## Graphical Abstract

**Graphical Abstract:**
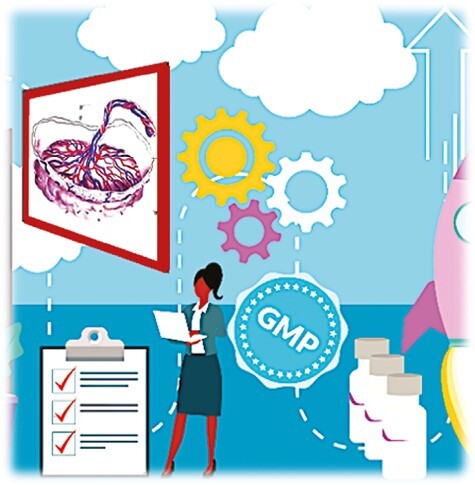

**Lessons Learned**

- Strategic projects and collaborative discussions among biomedical experts and clinical scientists are critical to translating novel treatments into clinical practice.
- Critical steps significantly limiting the translation of novel biological strategies into the clinic span from recruitment and collection of donor tissues to processing strategies and premise validation, validation of final product and risk management, up to distribution and reconstitution for final transplantation.
- Bio-engineered tissues and isolated primary stem cells from full-term placentae may produce a paradigm shift in interventional and regenerative medicine.
- Perinatal derivatives can offer direct correction or enhance innate regenerative capacity.
- Conversely to several allogeneic treatments, perinatal cells, and engineered tissues have been proven not to require any immunosuppressant regimen to support their beneficial effects.

**Significance Statement**

Advanced medical therapies require compliance with International regulatory agency guidelines or directives, as well as synergy between different professional figures. Strategic projects and collaborative discussions among European experts and clinical scientists in using perinatal derivatives generated a comprehensive and complete summary of recommendations for the generation of advanced medicinal products and bioengineered tissues. A revision of current regulatory frameworks resulted in instructions and recommendations to support product manufacture, testing, use, and marketing of such novel perinatal therapeutic catalog.

## Introduction

**Figure 1.**
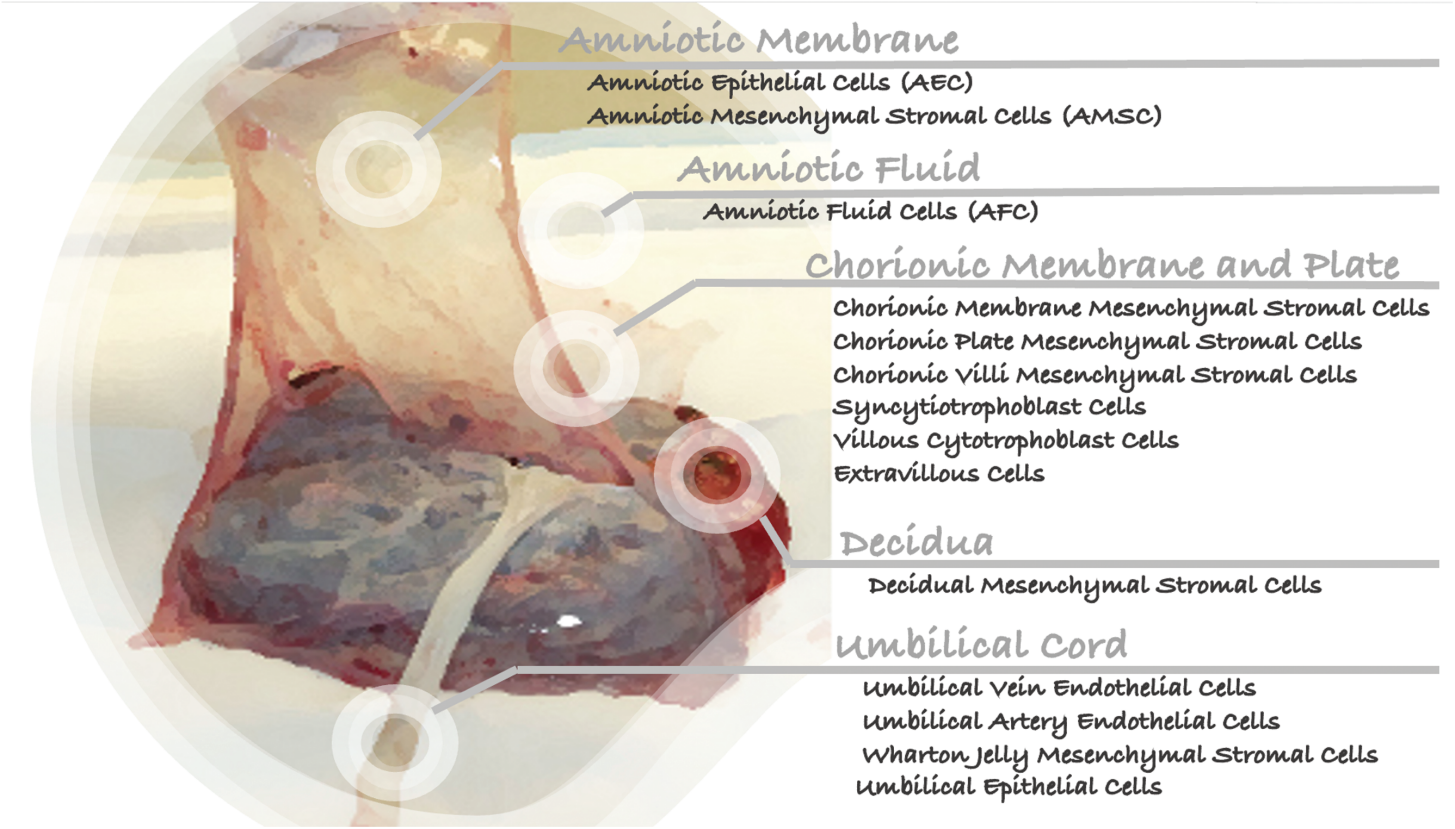
Schematic representation of different PnD products: in gray all the tissues and in black the most relevant cells potentially extracted from a full-term human placenta. Nomenclature has been revised and summarized accordingly to a previous publication.^1^

Perinatal derivatives (PnD) represent a new class of bio-engineered and advanced medicinal products gaining recognition and attracting growing interest as an unlimited source for multipotent stem cells, soluble mediators, and biological matrices. Under the umbrella of the PnD portfolio, we can identify intact tissues and mechanically isolated cells currently proposed as medicinal products (listed in Fig. 1, in gray and black, respectively).

The implementation of PnD in medical practice cannot be claimed as a recent proposal or innovative approach. The first documented use of perinatal tissue in support of skin regeneration is dated 1910 when human amnion membranes were implanted in more than 500 patients at the John Hopkins Hospital in Boston.^2^ Now, more than one century later, we can offer revised and optimized advanced treatments, based on PnD products generated and manufactured in compliance with Regulatory Agencies’ requirements. In addition to traditional tissue products generated by (minimal) manipulation of maternal and fetal tissues, during the past decades, optimized procedures have generated advanced technologies generated by mechano-enzymatic stem/progenitor cells isolated from both sides of the placenta. Seminal reports have highlighted that both intact cells and secreted mediators can treat and reverse critical medical conditions, including disorders currently suffering from limited or no effective therapeutic options. Similar to what happened in 1988, when the first perinatal product was implemented in clinical practice, with cord blood transfused into a patient with Falconi anemia.^3^ Nowadays, 35 years later, PnD translation into clinical practice for such a plethora of perinatal products requires strict compliance with International and Regulatory Agency regulations, in regard to donation, production, and distribution to the end user. Within the next few pages, experts and operators working on different levels of development and validation of PnD products have gathered under the umbrella of COST action. COST Sprint Action (CA17116)^4^ has attracted experts from 30 different European Countries, allowing leaders and experts in PnD at different levels of preclinical or clinical application to share and offer experience and know-how. Such a 5-year long activity allowed, among others, to compile this short overview of the minimal measures that need to be addressed in order to utilize such valuable resources for medical and veterinarian use. Such a set of preventive and quality measurements are strictly regulated by National and International Agencies and have recently been revised by the European Commission (resulting in the EDQM guide). Such documents accurately describe and detail all the requirements for collection, processing, and conditions for traceability and biovigilance, in addition to quality management and risk management, donor evaluation, and testing. And it is moving from such publicly available documents in addition to discussions between academic and commercial developers and experts in PnD technologies, we decided to generate this current work with the ambition to serve as a guide and support to developers, up to the final users.

The majority of the specifications here described apply in principle to all PnD products. However, some level of flexibility and a slightly different set of validation analyses can be applied to different PnD, depending on the nature of the tissue of origin and intended application. The measures differ with regard to the specific requirements for the product and the clinical application. While the established Quality Management System (QMS), as well as requirements for donor selection, donor testing, and collection of starting material, are equally valid for all PnD, some additional steps (eg, processing, product quality control, storage) vary among PnD products. An axiomatic example consists of the amniotic membrane: when such perinatal tissue is used as a matrix, both the manufacturing processes and regulatory criteria are different from amniotic cells when isolated after birth and intended to be used in allogeneic treatments. Amnion-derived cells, together with several perinatal cellular products, are classified as Advanced Therapy Medicinal Products (ATMP), and the adoption of stringent manufacturing and laboratory set of rules is crucial and mandatory. The adoption of current Good Laboratory Practice (GLP) and/or Good Manufacturing Practice (GMP) conditions for process development and scale-up of PnD products is critical for delivering a safe and effective treatment. To grant safety for recipients of human tissues, cells, and cellular mediators, strictly regulated and qualified criteria have been established for every single step, moving from donation and procurement to processing, preservation, storage, testing, and distribution of the final product to the end user.

**Figure 2.**
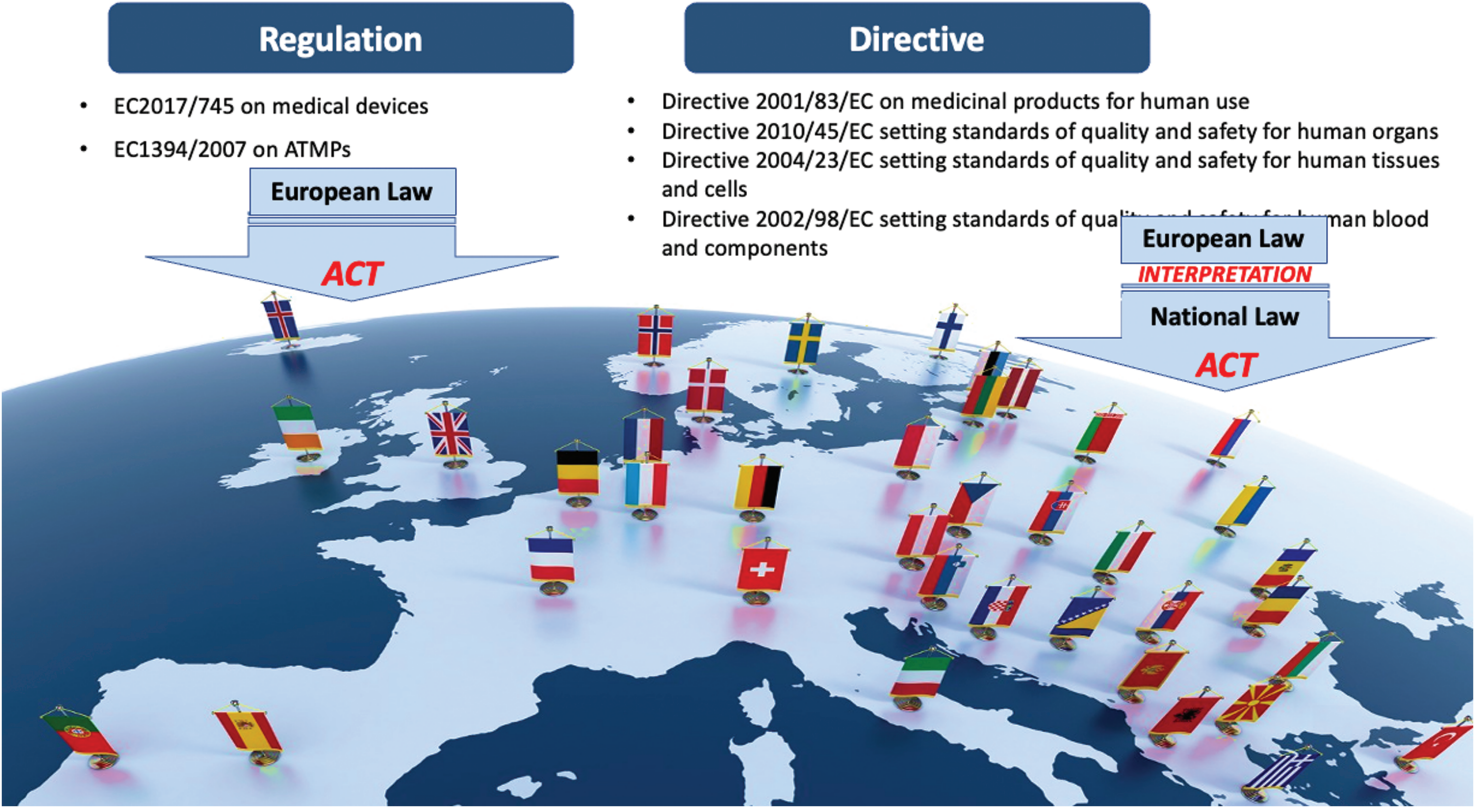
Current European directives and regulations pertinent to PnD and other advanced medicinal product generation.

Within the European Community, the growing need for innovative medicinal products and advanced therapies is inextricably linked to specific regulations and directives implemented for medicines and human-derived products. However, there is a clear and distinct difference between EU directives and regulations (Fig. 2).

A directive is a legislative act with a clear goal, enforced by all the active Members. Such act implementation mainly relies on the individual State Members, based on their own laws, and relying on National Agencies to define a path leading to these goals. Thus, specifications can differ from Country to Country. Conversely, a set of regulations are binding legislative acts, active at the European level. Such rules must be applied on their integrity broadly by every State, and a set of guidelines have been implemented to elucidate how such regulations should be applied and followed (eg, GMP guidelines). However, such a level of variability with related interpretation and enforcement clearly raises uncertainty and jeopardizes the adoption of therapeutically valuable PnD treatments with the European Community.

## Key Elements for the Production of a PnD Product

**Figure 3.**
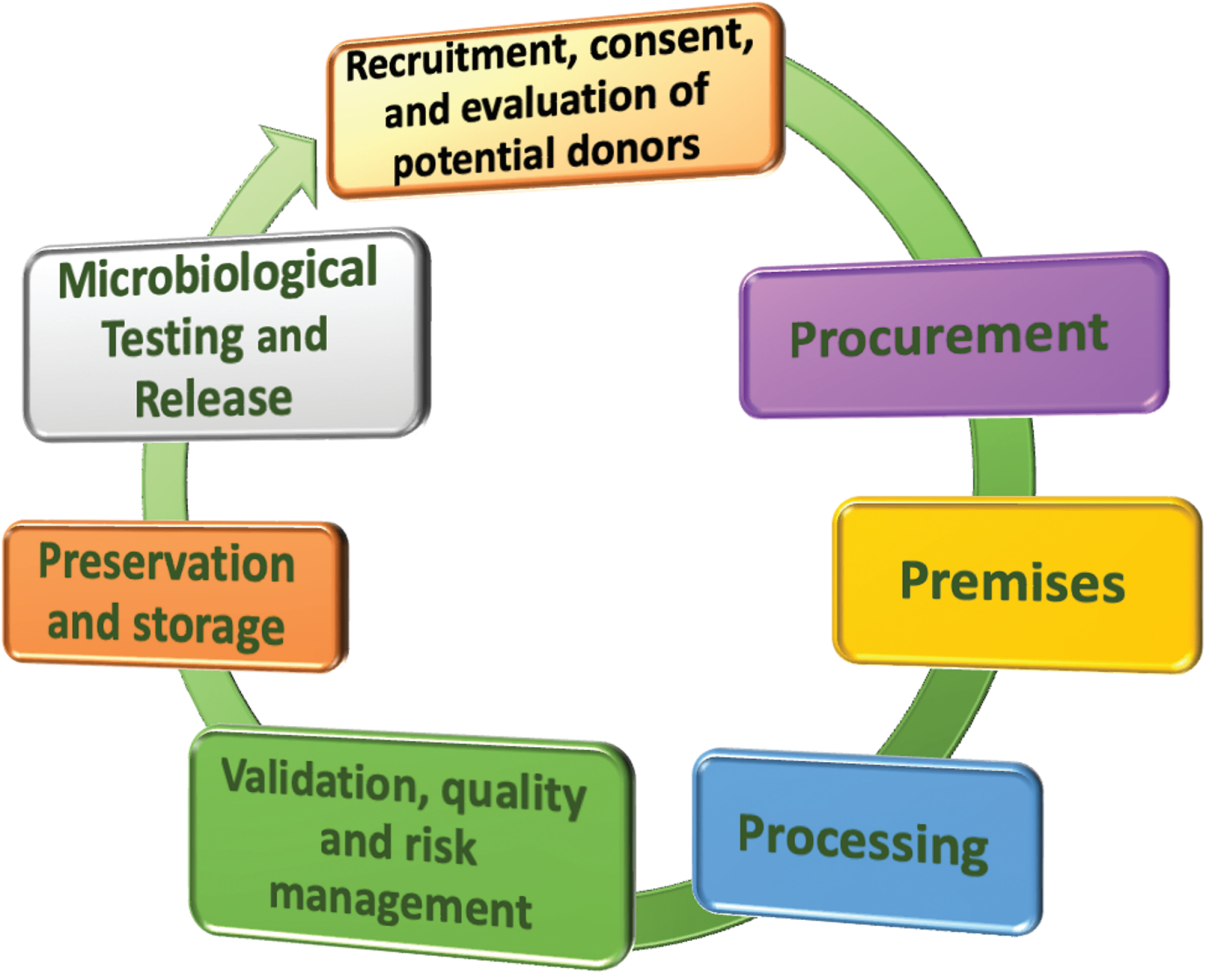
Key elements required to generate and quality PnD biomedical products in the order here described and discussed.

Within the next sections, we intend to outline and discuss the key elements (summarized in Fig. 3) required for the quality and safety production of PnD leading to clinical application. We will discuss and describe the production chain, starting from manufacturing facility requirements and biological source procurement (eg, donation), up to final product qualification and release.

### Recruitment, Consent, and Evaluation of Potential Donors

Tissue and organ donation have been the backbone for many medical interventions and resolutive treatments during the past century. Moving from blood transfusion to organ transplantation, the collection, and implantation of an allogeneic somatic tissue has sometimes raised ethical and technical problems. Undoubtedly, the biggest advantage of using placenta, collected at the end of pregnancy, is the complete lack of ethical issues and no additional risks for the mother or the newborn. Human placentae are commonly considered as waste material in Western Countries. Nevertheless, since perinatal tissues are collected from a living donor, many steps of donor recruitment and evaluation are facilitated. Interestingly, the human placenta is a hybrid tissue, mosaicism between tissues of maternal origin strictly interconnected to tissue and cells generated by the embryo during the 9 months. Thus, the mother, and the father in some jurisdictions, are called to serve as responsible persons to sign consent for fetal-derived material. The purpose of use for every part of the donated tissue needs to be fully declared in the informed consent and comprehensively explained to the donor(s). Concurrently, the donor(s) are allowed to provide social and medical history such as personal and behavioral information (including travel history), genetic disease, family and transfusion history, current medication, and status of recent vaccination. It is common practice to test donors for all high-risk pathogens, such as hepatitis A/B/C, HIV, and syphilis. Some medical centers perform additional screening on the cytomegalic virus, Epstein Barr virus, *Treponema pallidum*, *Neisseria gonorrhoeae*, *Chlamydia trachomatis*, and, if applicable, HTLV. Since 2020, almost every center adopted additional screening for SARS-CoV-2. All pathogen-positive deliveries are commonly excluded. Additionally, specific exclusion criteria applicable for PnD comprise significant viral, parasitic, bacterial, or mycotic infection (in particular amniotic infection syndrome); malformation of the fetus/newborn; endometritis; meconium ileus; premature rupture of the membranes.

The acceptance criteria for donor screening aim to minimize the risk of transmitting diseases to the recipient(s).^5,6^ A critical step in donor evaluation is represented by the identification of the donor(s) and validation of the consent. Within the European Community (EC), the selection criteria are described in Directive 2006/17/EC, Annex I-III,^7^ as an implementation of the previous Directive 2004/23/EC^8^ in terms of donation, procurement, and testing of human cells and tissues. Individual member states of the EC can set the acceptance criteria starting from the present Directive.

### Procurement

Procurement must be authorized by the competent Health Authority, and the collection procedure must fulfill the minimal requirements supporting the preparation of a clinical-grade product. All procedures should be carried out by qualified personnel, in compliance with Standard Operative Procedure (SOP) and risk assessment. The parameters that compromise tissue procurement are commonly in relation to the use of open/closed systems or dedicated restricted areas, the time between delivery and collection, the decontamination activities, and personnel training.^9^

In the frame of PnD collection, both the placenta and umbilical cord are collected by the medical staff at the obstetric unit, and the storage and transportation time should be minimized (the maximum recommended time is 24 h). The collection procedure for perinatal material is not dissimilar from the classical organ transplantation activity: tissues should be placed in a sterile, pre-labeled container or bags filled with an appropriate transport medium (or decontamination solution).^10^ The sterile packaging should then be placed in a suitable outer container for transport to the processing unit. For sterility reasons, in several processes, only the tissues collected after the caesarian section procedure can be accepted. Currently, there is no consensus on the supplementation of antibiotics in the transportation liquid or the temperature range between collection and delivery to the manufacturing unit. Commonly, if perinatal tissues are delivered and processed within 2–3 h after delivery, transportation at room temperature has been proven not detrimental and sufficient for the production of both tissue^11^ and cell products.^12^

### Premises

The facility where all the PnD products are generated must fulfill specific requirements and provide complete control of all critical areas of production. Specifications may vary according to the process performed or the type of product generated, and the intended use. To process ATMP and medical devices, GMP applies as detailed in the regulation EC1394/2007 and should be in compliance with the ISO standards 14644.^13^ The current legislation details the principles of cleanrooms or restricted barrier areas needed to be considered in a manufacturing process. For these types of products, the cleanrooms are classified as grade B rooms, equipped with a grade A compartment (commonly laminal flow cabinets) where the high-risk operations are carried out, supported by grade C or D areas where less critical procedures are performed (storage and documentation). Environmental parameters such as air pressure, temperature, and humidity must be monitored. To minimize the risk of contamination, a positive pressure is commonly applied in interconnected rooms, with a growing gradient from low- to high-risk areas. The appropriate air quality for handling human tissues or cells is usually equivalent to a grade A environment with a background of at least grade D. Recently, the “classical” concept of a GMP factory with personnel working within has been replaced by close system technology, where the starting material enter in a grade A “Isolator.” Such a complex device can be accommodated in a grade D area surrounded by personnel dressed with common laboratory coating.^14^

### Processing

Several reports during the past 2 decades greatly advanced the development of new interventional strategies and therapeutic applications using PnD products. All the birth-associate tissues and cells have undergone extensive description and characterization, leading recently to a consensus paper detailing the origin and criteria to identify such PnD products. To define various PnD beginning ingredients and final products listed below, we use such consensus nomenclature. ^1^ Essentially, 3 major PnD products are currently manufactured and tested in several clinical trials^15^: (1) intact tissue or decellularized tissue/matrix; (2) intact, viable cells isolated from different parts of the placenta or collected from the amniotic fluid and cord blood; (3) secreted mediators generated by ex vivo culture of perinatal tissues or cells. The term “processing” comprises all the activities required to prepare, manipulate, preserve, and deliver the final medicinal product. Every step carefully considers the sterility of the biological material, including repeated microbiological tests, in every aspect of the process, to document safety for the recipient(s) and operators working in the manufacturing facility. The quality control system begins with the procurement of tissues or cells in adequate conditions (packaging, the safety of biological tissues, and quality control of starting materials before procurement). The document must include the traceability of tissue (from the procurement phase until the final batch administration) and must be performed according to Directive 2006/86/EC.

When the final products are PnD tissues or matrices, the processing must maintain unaltered the physical, mechanical, and biological properties of the final product. Axiomatic is the process currently adopted to manipulate amniotic membranes. Processing of the amniotic membrane generally begins with the mechanical detachment of the fetal membrane from the underlying (fetal and maternal) placental tissues. Fetal membranes comprise both the amnion and chorion leaflets, interconnected by a jelly-like intermediate layer. The chorion may be discarded, and the amnion rinsed extensively to remove blood residues. In some procedures, both amnion and chorion tissues are processed concurrently, and the final product is generally referred to as an amniochorionic membrane. Such procedure is included and discussed in an EMA CAT document where the final product is clearly excluded from ATMP classification since the “does no longer contain cells or tissue.”^16^

But perinatal tissue is not limited to the main body of the placenta and surrounding leaves, the umbilical cord is probably historically the most used PnD material. As the main placenta organ, the umbilical cord can serve as a repository for multipotent cells (described later) as well as the extracellular matrix (Wharton’s jelly, WJ). To prepare ECM extract from WJ, the umbilical cord is frequently cut longitudinally, exposing the embedded blood vessels (one umbilical vein and 2 arteries) without disturbing the epithelium paving the external surface of the umbilical cord. The gelatinous layer of WJ is mechanically removed from the blood vessels and inner epithelium of the subamnion using a scalpel. During such processing, the membranes and WJ may be exposed to decontamination by surrounding the tissue in an antibiotic/antimycotic solution. The incubation temperature and time have to be defined and validated. Following the decontamination step and rinsing procedure, the WJ and membranes can be spread on fine MeSH gauze for easier handling, or on a suitable carrier membrane (eg, nitrocellulose), and, if needed, cut in pieces. Depending on the intended clinical use, one side of the amniotic membrane (epithelial and stromal layer) can be exposed face-up for further processing.

In compliance with the preservation method, matrix grafts may be sterilized or decontaminated by irradiation. The sterilization approach should be validated for the initial estimated level of bioburden.^17^ The resulting product should be then sealed in sterile containers and labeled. As previously mentioned, the longest storage time that such product can be preserved without major detrimental effects depends on the preservation method and is defined and validated case-by-case, and will be discussed later in the text.

**Figure 4.**
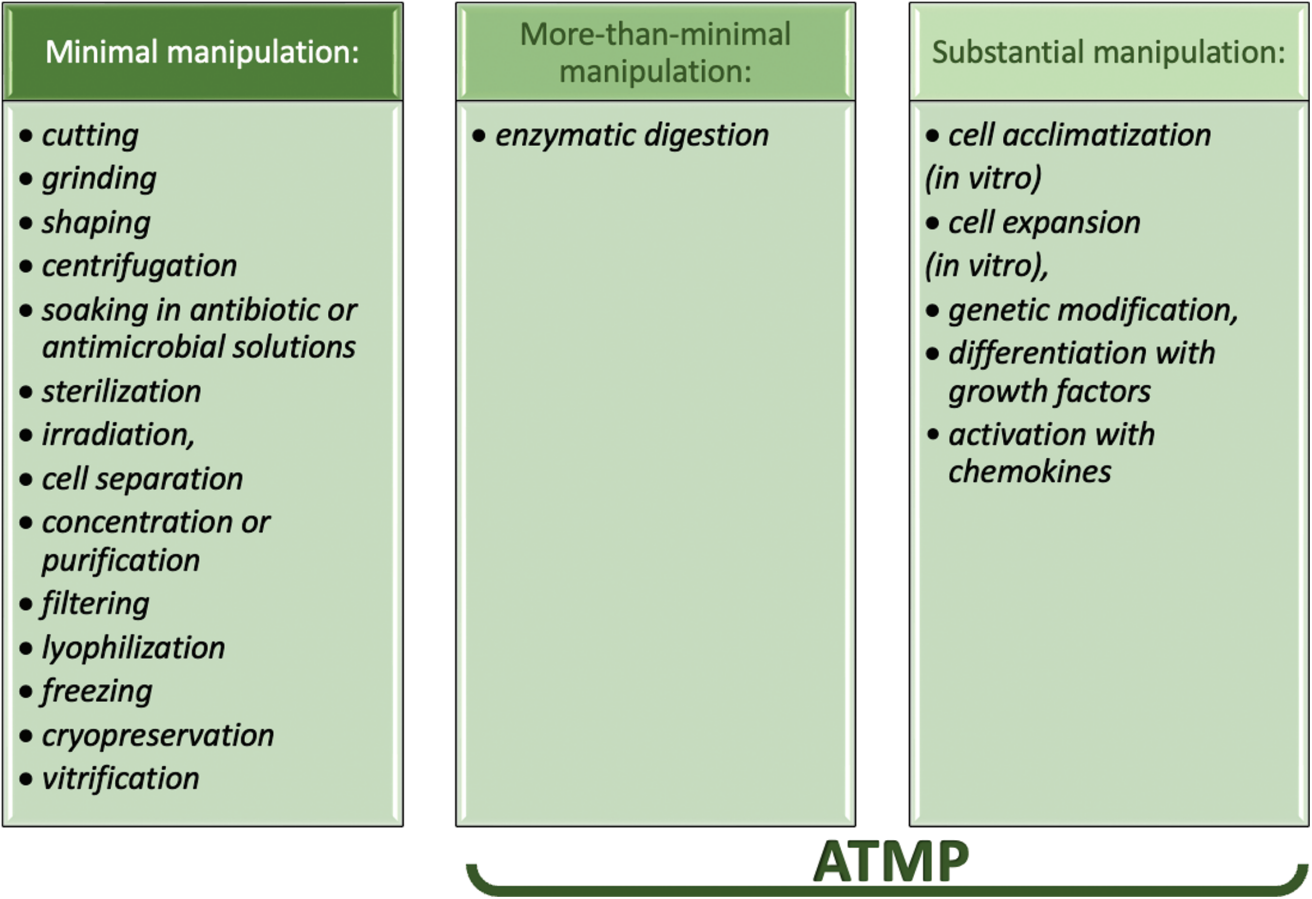
All the different laboratory procedures commonly involved in the preparation of biological medicinal products, subgrouped as minimal, more-than-minimal, or substantial manipulation according to EC1394/2007.

While clinical use of perinatal tissue is probably the first PnD product translated to the clinic (the first documented use of amnion membrane in medical settings is dated 1910),^2^ the largest use of perinatal cells is undoubtedly the infusion of cord blood cells and hematopoietic stem cell transplantation. In the latest 1980s, the processing of human cord blood was established and rapidly expanded in most medical centers worldwide. However during the past 35 years, it has become clear that full-term perinatal tissues may represent a promising and reliable source for multipotent stem and progenitor cells. The large-scale isolation and subsequent banking of stem cells have been the backbone of the emerging field of regenerative medicine. The processing of PnD cells comprises cell isolation, purification, and cryogenic preservation, spanning from minimal, more-than-minimal, and substantial manipulations. Cord blood and amniotic fluid products have been largely used and still not classified as ATMP since manufacturing procedures limitedly include manipulations listed in Annex I to EC1394/2007,^18^ and summarized in Fig. 4.

**Figure 5.**
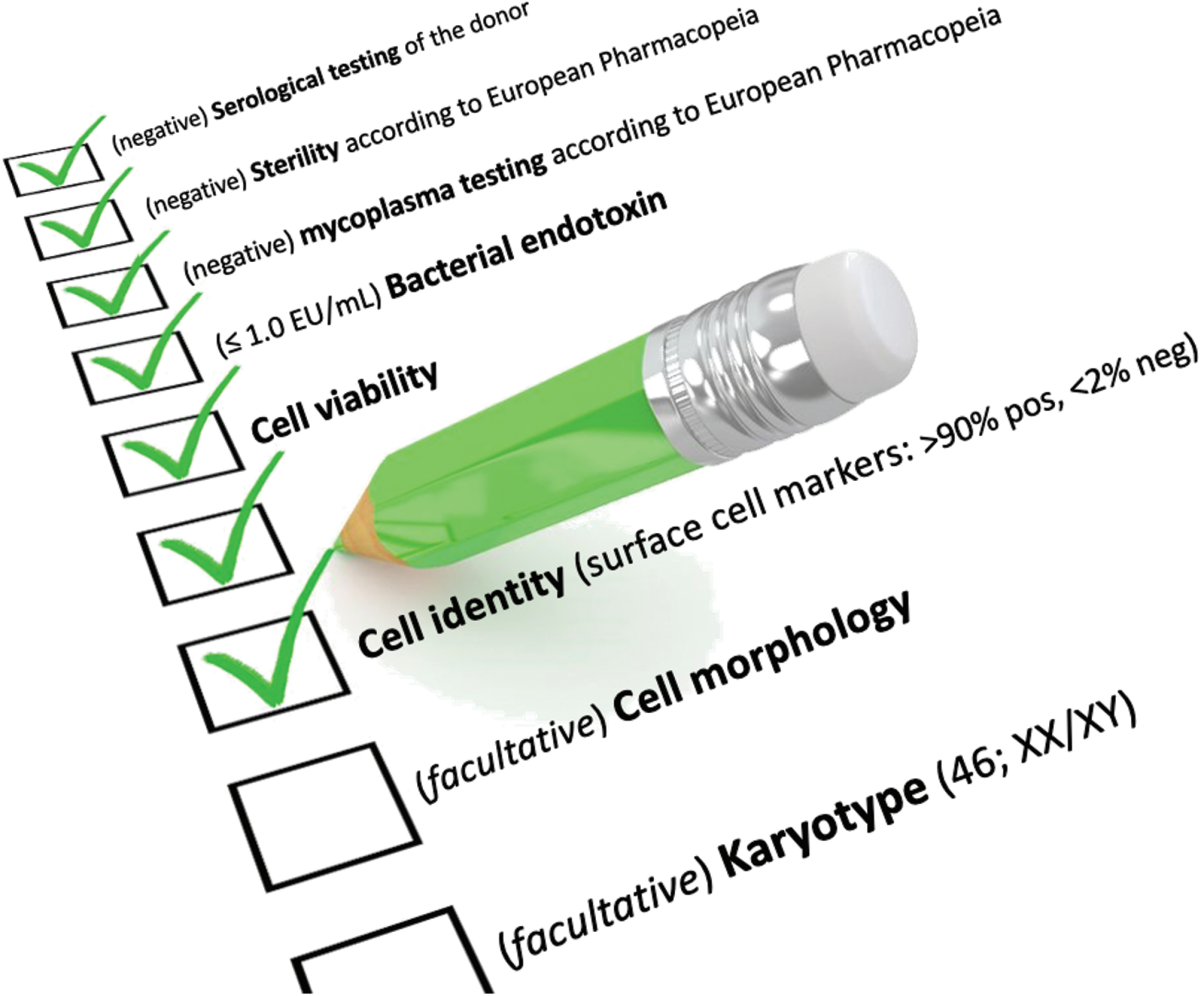
Schematic checklist for the release of a cell product.

Other perinatal stem/progenitor cells are ATMP and their processing should be in compliance with current GMP guidelines. Indeed, the release of cells following incubation with an enzymatic solution is considered as more-than-minimal manipulation, upgraded to substantial manipulation when cellular products are seeded in vitro. Robust pipelines and validated SOPs have been developed and standardized to generate clinical-grade batches of cells that have been used under hospital exemption applications or are under evaluation in several registered clinical trials.^1^ Commonly, the release of the final product is based on tripartite components, sterility-viability-identity, as required by International Regulatory Agencies (Fig. 5). Mechano-enzymatic procedures frequently result in heterogenic cell suspensions, and the presence of static cell markers on the surface of intact cells has been largely implemented to validate the identity of cell products.

Long-term maintenance or cell expansion in ex vivo conditions has been largely implemented in several processes to grant a sufficient number of cells to infuse in recipients, minimizing or avoiding multi-donor transplantation. However, standard culture conditions implementing culture media and supplements of animal origins had to be replaced and substituted, when possible, with GMP-grade reagents. The implementation of ECM proteins to support cell adhesion to in vitro conditions, as well as supplementation of a mixture of nutrients and growth factors, commonly supplied by fetal animal sera, has been recently replaced by human serological products or pools of platelets collected during blood donation and exposed to cryogenic lysing. Obviously, every medium supplement, as well as animal-free and GMP-grade reagents need to be validated and optimized. Genetic stability and transcriptional evaluations are also part of the validation process.

The ATMP classification is a voluntary procedure that can be applied at any stage of product development, before or after preclinical/clinical results have been collected. Such classification in Europe is conducted by the CAT, on request, and based on the information the developer is able to provide, based on processing and product specifications.

While the recommendations on the classification provided by the International Regulatory Agencies are not binding, such classification may support researchers and developers to harmonize and get their technologies recognized (with granted relevant services and incentives offered) by the EMA.

### Validation, Quality, and Risk Management

To fully ensure the safety and quality of a cell or tissue preparation, the entire process must be under the supervision of a Quality Management System (QMS). The QMS is set out in a regulatory document detailing all the activities in relation to process manufacturing, spanning from the identification of a potential donor to the final batch preparation for clinical use.^19^ Some elements need to be present and described in QMS documentation and subjected to continuous maintenance. Such critical elements are summarized and described in Box 1.

**Box 1: critical elements included in the QMS document**

**Table:** 

1	*Quality control* (QC) testing list executed during the manufacturing process such as verification steps, sampling, and testing applied to materials, processes, and the final product. The QC results must be acceptable and within the range stated in the QMS.
2	The ***quarantine*** status of starting materials and/or final product requires that all quality-control tests and checks have been conducted. Instead, the concept of the *release* status of final product is confirmed by 2 consecutive steps: the acceptance criteria of the donor and compliance of cells or tissue defined in the product specifications. Notably, the concept of “Quarantine and release” is not applicable in autologous treatment.
3	The ***change-control*** procedures that should be carried out prior to the implementation of a revised/new process. Written procedures describe the action to be taken if a change is proposed.
4	The full ***traceability*** of all materials, reagents, and equipment that come into contact with tissues and cells. All the **deviations** reported during the manufacturing process must be documented, carefully investigated, and managed in a timely manner. Any reagent known to potentially cause adverse reactions in the recipient should be reported and described.
5	The ***internal*** and ***external audit,*** are essential tools for ensuring compliance with the quality system and for supporting continuous quality improvement. All audits should be documented and recorded (especially external audits for designation of ISO certification).

In order to reach the most important requirement (quality) is mandatory to (1) define the regulatory context in terms of donation, procurement, testing, processing, storage, distribution, and import/export activities for tissues and cells; (2) use a robust QMS in compliance with legal requirements (in some cases use the specific tool in relation with different types of tissue or cell); and (3) obtain the authorization for specific activities.

The validation activity certifies that all critical aspects of the establishment’s operations relieve patients of any risks and are in compliance with the clinical scope. Such validation is commonly divided into 2 components: qualification and process validation. The term “qualification” applies to each part of the process and assets (including cleanrooms, facilities, equipment, materials, and operators). The qualification must be conducted before the first use, and revised periodically, or when significant changes in the SOP are introduced.

The process validation can be subdivided into prospective validation (performed when a new process is initiated or when required by local legislation); concurrent validation (performed during the process of manufacturing); and retrospective validation (performed once the process is completed).

These activities’ objective is to identify any critical aspects through a series of controlled tests. Consequently, the activity requires a full understanding of the risks associated with any critical process and must be described in a risk matrix documentation. Such an approach includes the identification of the possible risks for the process in the products and also in the personnel involved and then evaluated from a quantitative risk analysis. Updated risk-mitigation strategies need to be developed and implemented to protect both donor’s materials, principally the recipient(s). Risk analysis and mitigation strategies need to be performed at any process change implementation or when new documentation is integrated. Corrective And Preventive Actions prevent any occurrence in the future.

The validation policy should comprise the process design phase, where specific expertise and knowledge for the proposed process are required. During the validation phase, the process control strategy must be implemented, and all the assets involved, such as equipment, utilities, suppliers, and transportation, need to be qualified before proceeding with the process validation. Step-by-step subsequent validation should be carried out.

**Figure 6.**
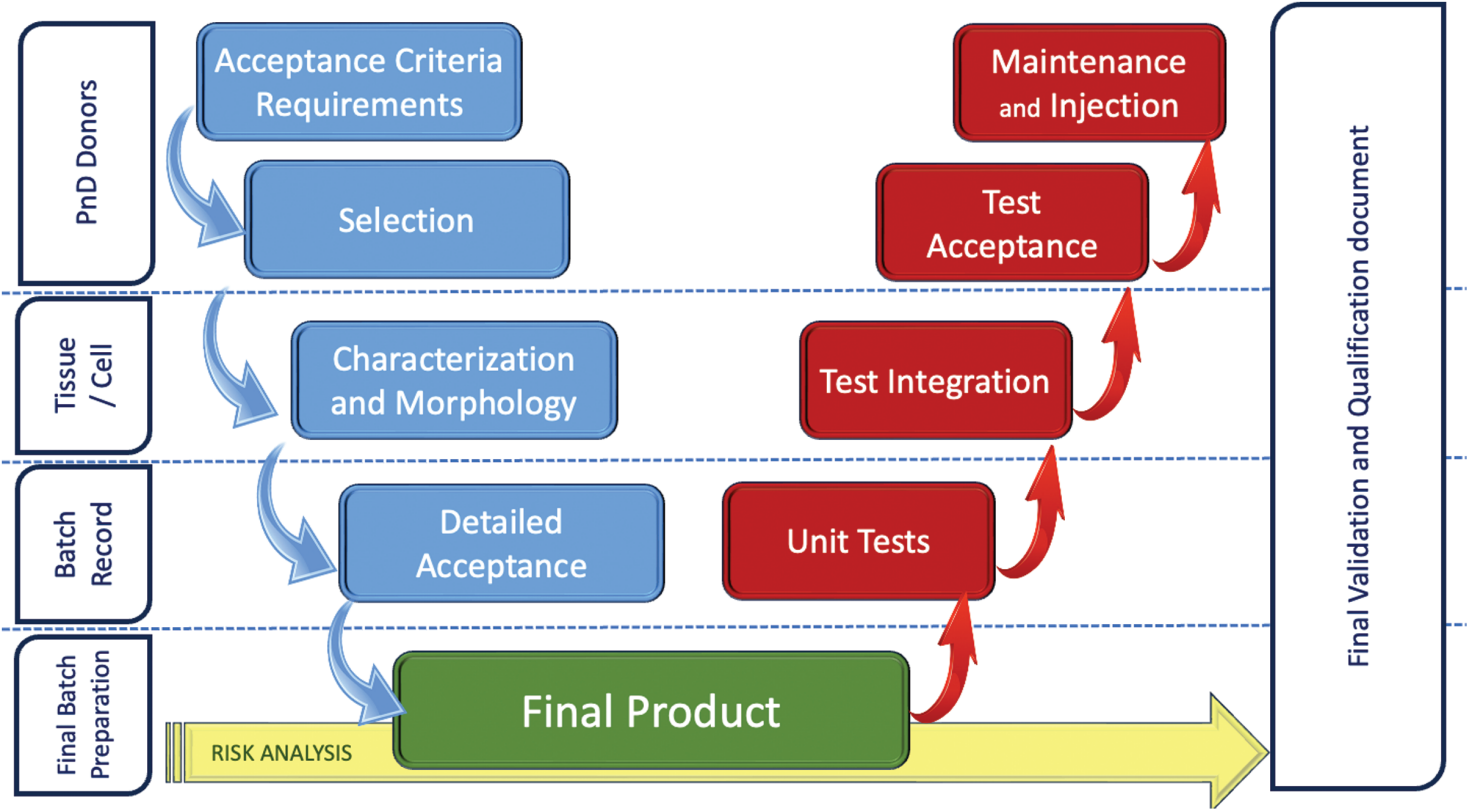
Validation plan strategy.

The implemented methods and the acceptance criteria should be approved by the establishment management and documented before qualification or process validation is initiated. Such a document is frequently called a “validation plan,” and a model is depicted in Fig. 6. Such validation should be performed by trained and competent personnel, and the results in compliance with acceptance criteria. All these elements articulate the QMS document. The process and policy (planning, executing, and recording validation) should be documented in written procedures and assembled in the “Validation Master Plan” documentation.

### Preservation and Storage

Once the PnD product is generated, the storage and preservation of the final batch without detrimental effects are also a critical part of the manufacturing process. It is critical to optimize and validate preservation techniques for the intended use. The selected technique should allow the retainment of the critical properties (eg, structural integrity) for the longest possible storage period. Similarly to what is described in the preparation methods, the storage conditions should also be analyzed and optimized for the starting material and final treatment. Consequently, different preservation procedures, spanning from normothermic, hypothermic, or cryogenic conditions, have been tested and described. Tissues, cells, and secretomes can be preserved by different techniques including, but not limited to, refrigeration/freezing, cryopreservation, vitrification, drying/lyophilization, or glycerolization. Once in the frozen state, “off-the-shelf” PnD batches can be transported in liquid nitrogen dry shippers (or equivalent) to Transplant or immunology centers.

#### Tissue

Refrigeration or hypothermic preservation is frequently applied to intact tissues and organs and implies the reduction of temperature in the surrounding transportation liquid to minimize cell metabolism and prevent necrotic and apoptotic events. Cryogenic preservation is used as a pre-processing step, or as in-processing storage for non-viable tissues. However, cryopreservation is more frequently implemented to store the final product awaiting release for transplantation. Cryopreservation may disrupt tissue and cell integrity.^20^ Hence, the selective choice of a freezing method should consider the end user’s needs and clinical applications.

PnD can be stored in hypothermia (<20 °C), low (deep frozen at −60 to −80 °C), or at ultra-low temperatures in vapor or liquid nitrogen (−140 to −196 °C). Cryopreservation (or low/ultra-low temperature preservation) is a process where the biological and structural functions of tissues or cells are preserved in sub-zero temperatures, lower than −140 to 196 °C (storage in liquid nitrogen or vapors), respectively.

The preservation of the PnD matrix (amnion/chorion membrane and WJ) requires the packaging of the final product, followed by low (−80 °C) or ultra-low temperature storage after controlled-rate freezing.^21-23^ Long-term storage (up to 5 years) has been reported. However, storage at higher temperatures (−80 °C) has also been successfully described when a cryoprotective agent (CPA) is supplemented (maximum storage time, 2 years). In such tissue products, where maintenance of cell integrity and viability is not strictly required, the PnD product can be preserved without the addition of CPAs.^24^

Another approach largely used to generate PnD matrices involves overnight heat drying in an oven at 40 °C, or alternatively air-dried at room temperature. Once dehydrated, the PnD product can be packed and eventually sterilized by irradiation. The long-term storage of such a product is also carried on at room temperature.^25-27^

A third option for a PnD tissue product involves lyophilization. Lyophilization (or freeze drying) involves the dehydration of the product. Such an approach requires reducing the temperature as well as the pressure to allow water to sublimate directly from the solid phase to the gas phase. The PnD matrix is rapidly exposed to low temperatures, 50-80 °C below 0 °C (possible with lyoprotectants). Then, the final product can be then vacuum-dried and water once again extracted through sublimation, leading to a final water content not higher than 5-10%. Lyophilization allows storage at room temperature and prevents tissue autolysis. Following the package, PnD matrix grafts can be sterilized by irradiation and stored at room temperature.^27^ Recent studies support the implementation of lyophilization procedures to retrieve viable cells as well if an optimized process with lyoprotective agents can be optimized.^28^

PnD tissues have been also reported efficiently preserved in glycerol. Glycerolization is a preservation method benefitting also from the antimicrobial properties of high concentrations of glycerol. Benefitting from the fact that glycerol permeates slower than water, an initial efflux of water is commonly achieved by the addition of glycerol. However, as glycerol begins to permeate, water may re-enter the tissue. Once glycerolization is completed, the final water activity is ~0.3, a value known to reduce lipid peroxidation and other degradation reactions. Rather than dehydrating the tissue, as is commonly assumed, it has been shown in wound healing applications that glycerolization results in the sequestration of water.^29,30^ Typically, 85% glycerol is used to preserve fetal membranes for up to 2 years hypothermically.^31^

#### Intact Cell

Human cells can be stored at room temperature, or hypothermically (similar to what is currently performed with solid organs). For instance, sub-confluent cultures of MSC have been stored at 4 °C for 2-4 days. Then, such products can be recovered by transferring at 37 °C for a few hours. Such a procedure has been reported as efficient in recovering viable cells with typical surface marker expression, proliferation capacity, and osteogenic potential.^32^ During such hypothermic storage, the supplementation with chemical modulators (such as resveratrol and salubrinal) has been proven beneficial to improve cell viability.^33^

After isolation and/or expansion, the cells can be frozen with the addition of a cryoprotectant which will preserve them from damage caused by the formation of ice. The allocation of cell products is greatly facilitated by the possibility of long-term storage and distribution in a cryogenic state. However, such cryogenic preservation may generate detrimental effects on the intact cells, where the ice crystal formation and the increase in the volume of the cytoplasm frequently irreversibly damage the cells. To prevent such effects, several CPAs have been tested and implemented.

Intracellular CPAs have low molecular weight: dimethyl sulphoxide (DMSO), dimethylacetamide, ethylene glycol, glycerol, methanol, and propylene glycol 1,2-propanediol. Extracellular cryoprotective agents are oligosaccharides (ie, Trehalose) or have a higher molecular weight (ie, hydroxy ethyl starch, albumin, and polyvinylpyrrolidone). Intracellular CPAs are considered the more effective type; they penetrate into the cell and prevent ice crystals. However, due to their high penetration capacity, such penetrating molecules result in a high level of toxicity at physiologic temperature. Among intracellular cryoprotectants, DMSO is by far the most used. A final amount equal to 5%-15% is largely used in clinical protocols, and GMP-grade commercial solutions, supplemented with different amounts of DMSO are commercially available.^34^ Mutsenko and coworkers proved that electroporation could improve preservation by introducing sugars into hUC-MSCs (sucrose, trehalose, or raffinose) that are normally extracellular CPAs. On average, more than 80% of cells were recovered after thawing^35^.

The cooling rate has also been reported as critical to preserving PnD products. To generate a progressive decrease in temperature, cells are placed in a freezing device (such as a controlled-rate freezer) that gradually reduces the temperature.

Another promising strategy for reaching ultra-low storage temperature for long-term cell preservation is vitrification. One of the major obstacles to successful vitrification is the possibility of generating rapid cooling and heating rates to minimize vitrification/devitrification and crystallization/recrystallization-induced damages during cooling and rewarming at thawing, generated by intracellular ice formation. The usage of magnetic induction heating of magnetic nanoparticles to enhance rewarming was successfully used to overcome this hurdle^36^ for human umbilical cord MSC (hUC-MSCs) preservation, using a culture medium containing 1,2-propanediol, Ethylene Glycol, and trehalose as cryoprotectants.

**Figure 7.**
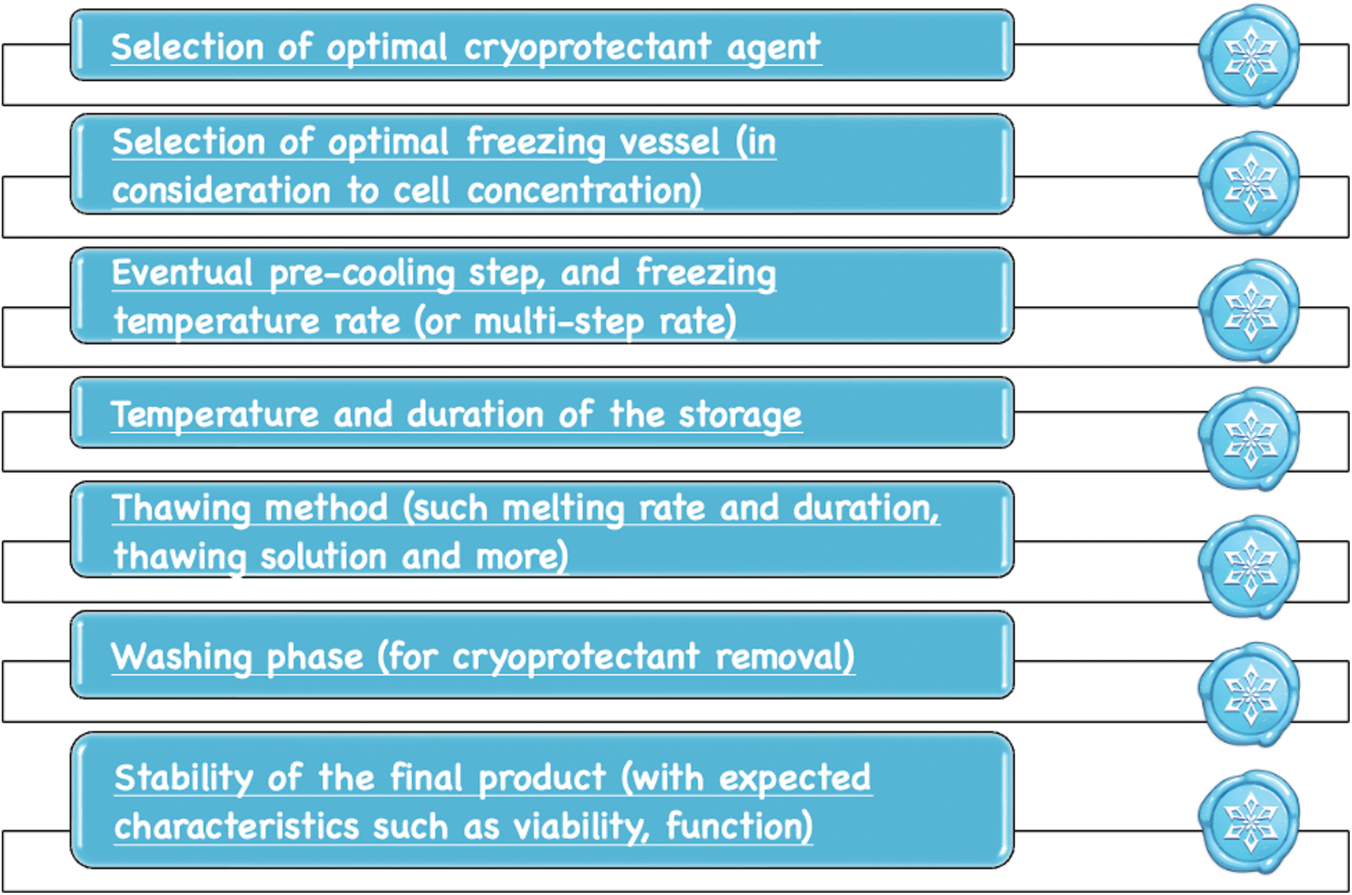
Critical elements for successful cryogenic preservation of biological products.

The validation of cell preservation should consider all the critical points summarized in Fig. 7. However, such a method of preservation is not risk-free, and the thawing step is frequently accompanied by a loss in viability and cell recovery, or sometimes, a reduction/erase in critical functions.

Relatively few studies have specifically studied the effects of cryogenic preservation on PnD products.^37^ The extrapolation of data obtained on adult cells to perinatal cells, even of the same cell type, is not obvious. An abundant amount of data is available, established from MSCs isolated from adult tissues, adipose, or marrow, which presumably could also be applicable to UC-MSCs. In a study with marmoset monkey cells, it was shown that the optimal cooling rate, and accordingly the post-thaw viability obtained with the same freezing protocol, were different between MSCs isolated from amnion and bone marrow.^38^ Moll et al showed in the clinical setting that fresh BM-MSCs have an improved efficacy compared to freeze-thawed MSC, possibly because thawed MSCs can experience a substantial heat shock response related to freeze-thawing.^39^ This phenomenon is sometimes called “the cryo stun effect.” The thawing procedure led to an alteration of the immunosuppressive effect of MCS, in an unsuspected way if it is not specifically verified because the phenotype itself is not modified.^40,41^ Accordingly, a high freeze senescence may correlate with poor post-thaw function in MSC samples^42^ and with an exhaustive number of freezing steps.^41^ Moreover, cryopreserved MSC is susceptible to T-cell mediated apoptosis^43^ or secrete lower levels of growth factors.^44^

Cryopreservation or vitrification in MSC preservation issues was recently reviewed in detail.^34,45,46^ Cryopreservation should not be considered successful based on post-thaw cell viability alone. Studies validating the preservation of cell viability and identity are strongly recommended for any PnD cell product,^37^ eventually coupled with biodistribution studies in support of efficient engraftment in the target organ.^47^

Differences between fresh and thawed cells might be limited but significant and should not be minimized. Indeed, cryopreservation could be an uncontrolled variability factor that could explain discrepant results, for example between pre-clinical models of bone marrow-derived MSC effectiveness and negative outcome of a clinical trial for the treatment of steroid-resistant acute graft-versus-host disease.^48^ Because of the disappointing outcomes obtained with clinical trials that used cryopreserved MSCs, some authors recommend that cryopreserved MSCs should be allowed to recover after thaw (an “acclimatization” time, for instance, 24 hours) and reach confluence.^45^ To avoid such substantial manipulation, another option would be a series of washes followed by centrifugation steps, such a strategy would clean final products from cell fragments or apoptotic bodies and enrich in desired cell product.^37^

From the perspective of biobanking, the cryopreservation of an entire tissue, for instance, the umbilical cord, for the subsequent isolation of the cells and their expansion, is an interesting strategy. Interesting studies have supported the possibility of isolating and cultivating MSCs from neonatal frozen tissues, quite similar to those obtained from fresh tissues (for review see^49^). Puzanov et al showed that cryopreservation of primary HUVEC does not affect the physiological features of such umbilical endothelium. Such an approach could support the efficiency of biobanking logistics,^49^ but in practice, does not solve the difficulties of preparing the cell product at the right time for clinical use.

#### Secretome

The cell secretome is composed of proteins, enzymes, and miRNA, released in the surrounding compartment as soluble mediators or embedded inside extracellular vesicles (EV) vehiculating such signaling even to long distances. EV is a generic term to describe vesicles of different sizes and composition, commonly grouped into 3 categories: (1) exosomes (30-150 nm in diameter), formed from intraluminal vesicles; (2) microvesicles (50-1000 nm in diameter), released by exocytosis; (3) apoptotic bodies (from 500 nm to 5 μm), plasmic membrane budding. In particular, exosomes play pivotal roles in cell renewal, immune surveillance,^50^ tissue repair,^51^ blood coagulation,^52,53^ and inter-cellular communication. The production of EVs as biomedical products initiates with the collection of cell supernatant after 24-48 h of culture, followed by a process of purification (such as serial ultra-centrifugation, tangential flow, and size exclusion chromatography). The scientific community is still debating and investigating the optimal conditions to preserve cell secretome integrity and bioactivity, including lyophilization and low-temperature preservation. No consensus has still reached. Nevertheless, it is necessary to determine and validate the optimal storage conditions for biological products (tissue, cells, or secretome) to verify, before the start of a clinical trial or the commercialization, their impact on the expected functionality of the cells. If necessary, consider allowing time for acclimatization after thawing.

### Microbiological Testing and Release

Similarly to what is described in the section Recruitment, Consent, and Evaluation of Potential Donors (donor evaluation), a macroscopic examination of the starting material (ie, fetal membranes) can be easily performed during procurement and processing. Any visible pathological alterations or damages within the structural integrity of the tissue can lead to the exclusion and rejection of such a donor. The size of donor material may also represent a valid method to prevent the processing (an inadequate graft size is also a valid reason to stop any further manipulation).

The European Pharmacopoeia (Ph. Eur.) represents the primary source of information for quality standards and requirements for medicinal products and drugs, including their ingredients, in Europe. Such a document provides the scientific basis for product QC, supporting the pharmaceutical and biotech companies, as well as the healthcare system. Such standards are legally binding requests and directives defined by the Council of Europe Convention on the Elaboration of a European Pharmacopoeia and in European Union and national pharmaceutical legislations. Indeed, every medicinal product needs to fulfill the sterility requirement, and PnDs are not an exception. Both ATMP and minimally manipulated products are processed under strict aseptic conditions, to prevent contamination, and finally tested for endotoxin level or mycoplasma presence, within all other sterility tests.^54^ One representative sample or a satellite vial (in the case of cryopreserved products) is periodically withdrawn from the batch and tested for sterility and quality. The representativeness of the sample is based on the final volume and homogenization of products. All the considerations above apply to ECM-derived products or cellular ATMP. Furthermore, in tissue products (ie, amnion membrane), representative samples are obtained with the same technique largely used for organs: bioptic material or surface swabs are sufficient to determine the sterility and integrity of the final product. However, the final product is not the sole sample commonly tested during processing, bioburden testing should be performed on transportation liquid, and washing solutions once got in contact with the PnD material, as well as post-sterilization or decontamination. Anaerobic, aerobic, and fungal testing for cell or tissue samples are the most direct qualitative measures of microbiological contamination.^54^ The presence of antibiotics during transportation can affect the sensitivity of the tests (leading to false negative results). Germ detection methods must be validated in the presence of contaminants (ie, albumin, or ECM proteins), known to affect the detection probes. The microbiological method suitability must be in compliance with the European Pharmacopoeia and validated for different bacterial, fungal species, and other microorganisms.

For sterility testing, the direct inoculation method is suitable for solutions and tissue samples. Alternatively, Ph. Eur. 2.6.1 allows filtration with filters with a nominal pore size equal to or lower than 0.45 μm,^55^ with proven effectiveness in retaining microorganisms. After filtration, the filter is transferred to an agar medium for incubation. During incubation, a minimal number of intermediate and one final reading should be recorded.

Microbiological control of products presenting a risk of contamination by environmental germs must be incubated at 2 different temperatures in order to allow the growth of a wide range of microorganisms. Classical sterility testing implies 2 broths, assessed macroscopically for evidence of microbial growth ideally daily; at least one intermediate reading and one final reading should be made. The incubation of soya-bean casein digests medium in aerobic conditions at 20-25 °C, allowing the detection of fungi and bacteria. The incubation in fluid thioglycollate medium, in anaerobic conditions at 30-35 °C, allows the detection of anaerobic bacteria. Automated culture systems generally take very regular readings and report microbial growth as soon as it is detected via CO_2_ production. Broth bottles can be inoculated with a volume of up to 10 mL and sometimes contain resin or activated carbon neutralizing microbial growth inhibitor additives. Incubation should last at least 7-10 days for automated culture systems, extended up to 14-21 days in case of slow bacterial growth, or for classical sterility testing. Precautions should be taken against microbial contamination during a test.^55,56^

Bioburden is defined as the number of microorganisms on a surface or volume,^54^ determined either by membrane filtration or by direct plate counting methods.^57^ Common methods for automated and sensitive microbiological tests consist of consumption/production of ATP, turbidimetry, cytometry, PCR, and more (refer to^57,58^ for a complete list of approved technologies)

The presence of mycoplasma^59^ and bacterial endotoxins^60,61^ can be detected in PnD products (such as in processes involving products of animal origin): both mycoplasma and endotoxins alter cell metabolism and activities in vitro, and such contaminations should be carefully avoided since they may severely compromise the safety and efficacy of the PnD graft (particularly in immunocompromised recipients). Mycoplasma particles are not retained by filtration, and endotoxin binds to the cell membrane and leads to cell lysis. Endotoxins are frequently detected via the Limulus Amebocyte Lysate assay, but can also be detected via biomolecular approaches, according to European Pharmacopeia.

In general, products found contaminated should not be released unless their clinical relevance justifies such exemption. Eventually, the identification of the germs and antibiograms allows a risk assessment based on such microorganism pathogenicity, leading to the possibility of administering an antimicrobial supplement to the final product or recipient. If the process involves terminal sterilization of the product within the final container, parametric release based on process data (and not on final microbiological tests) is still acceptable, if each critical step (including sterilization) is validated. If antibiotics are used in any step, before or during processing, such critical information must be indicated on the package leaflet, to acknowledge the final user about possible adverse events or preventive measures to adopt after use.

## Recent Advances in PnD Preclinical and Clinical Studies

During the past decade, a growing number of clinical trials using PnD products have been registered,^15,62^ and the (scientific and commercial) interest in such novel and promising therapies has literally “exploded.” Due to the presence in both scientific literature and market portfolio of such a plethora of treatments, where different parts of the human placenta were dissected and utilized, a group of European experts and specialized operators felt the need to share a common experience and discuss past and current experience under the umbrella of COST action. Approximately 5 years ago, the COST Sprint action (CA17116) offered the possibility to gather together a growing amount of experts from several European Countries, leading to the generation of different documents and guidelines published in open-access journals in support of manufacturing and exploitation of PnD. The present work represents one of the latest documents compiled by such experts and operators. With tissue and cell manipulated to generate new treatments and potential new therapies for orphan diseases, a group of clinical experts and manufacturers revised current European legislative frameworks to offer a revision and indications on such a complex set of rules and requirements for PnD biological product manufacturing and commercialization. International and European regulatory frameworks have laid down instructions and recommendations to monitor product manufacture, testing, use, and marketing of these new biological products. A revision of current rules and requirements across individual European countries may support and guide the generation of a novel PnD therapeutic catalog. The revised and described key elements, framed within the European Community, have no limited value within the European Medicines Agency, but they will definitely cover requirements and requests supported by other International Agencies to grant the path of product development and associated requirements clear and transparent.

As a consequence of human origin and PnD product complexity, the existing preclinical models are sometimes ineffective in predicting the clinical effects. The lack of appropriate animal models is not a reason to prevent or block new therapeutic approach development, particularly in areas of high therapeutic need. We have frequently experienced data generated in preclinical models raise concerns rather than confirm and support their clinical translation. Rodents and lagomorphs are largely diffused preclinical models, easy to use, and characterized by limited life span and high proliferative rate. Nevertheless, large animals are frequently implemented in a second step, depending on the product type and indication. However large animals are difficult to use and economically challenging. The use of preclinical models has been recently revised and in vitro alternatives have been largely recommended and requested by Regulatory Agencies. Preclinical results may generate a false sense of safety or risk, heavily impacting the diffusion and market approval of such a novel product. Nonetheless, preclinical validation based exclusively on ex vivo models is still considered high risk. The transfer into humans for the first clinical trials is offered with an extra-cautious approach, where product characterization and analysis of potency may imply a high level of complexity, much higher than other conventional biochemical molecules. Indeed, the manufacturing process and distribution represent 2 of the pillars supporting the novel treatments promising to revolutionize regenerative and personalized medicine.

Despite studies on safety, carcinogenicity, or toxicity (including repeated dose) being in principle needed and similar to conventional pharmacological treatments, a biological product may lead to long-lasting persistence and exposure to biological effects. Such effects may vary with time and biodistribution. The distribution and long-term engraftment, with relevant modifications in the ectopic site of action, may gamble clinical outcomes and expected results. The limited knowledge of the mechanism of action offered by biological products can exacerbate the lack of confidence in clinicians, reluctant to apply new treatments. All the aforementioned concerns form the basis for the “risk-based approach,” as defined by the Committee of Advanced Therapies (CAT), a specialized group within the European Medical Agency (EMA), revising any new ATMP.

The term “cellular therapy” identifies a modality of medical treatment in which pharmacological molecules and products are replaced with intact cells or cellular mediators. Examples of classical cellular therapies that have been registered and translated into clinical practice during recent years include bone marrow or peripheral blood stem cell transplantation and, more recently MSC isolated from different tissues. Similarly, it is undoubtedly the 2 most diffuse and largely known products generated by PnD are membrane derivatives and MSC isolated from different parts of the placenta or amniotic fluid. Mesenchymal stromal cells, for a long time (and sometimes still), mislabeled as mesenchymal stem cells, are multipotent cells with excellent proliferative capacity (preserved ex vivo). Stromal cells are present in the connective tissue or organ, therefore so MSC can mean that too. These cells can be isolated theoretically from every somatic tissue (fat, muscle, dental pulp of deciduous teeth, and more), but bone marrow was the primary source for such MSC. Bone marrow-derived MSCs are still considered the gold standard for several medical applications. Perinatal tissues have also been largely studied and confirmed as a valuable source of high-quality MSC. Both the umbilical cord and blood, as well as the maternal and fetal layers of the full-term placenta have been proven excellent sources of MSC, with some level of differences and enhanced features when perinatal cells have been compared to somatic cells (agreement on such regard and properties is still pending). Approximately 30 years ago, Dr. Caplan coined the term “mesenchymal stem cells,” based on these cell multipotencies and highly proliferative capacities.^63^ However, recently, he publicly revised such multipotent cells as stromal medicinal products, whose paracrine effects, rather than differentiation capacity, represent their most prominent mechanism of action. The exact mechanism(s) of action for different MSCs is still unknown, but it has been broadly reported that these bioactive cells can home in sites of injury and support the innate capacity to regenerate. Or, MSC can secrete active mediators and trophic factors to induce regeneration. During the past years, several experts have highlighted that MSC action serves as a promotor or adjunct treatment in support of tissue-resident progenitor cells. Tissue progenitor cells may actually serve as real fabricators for new tissue, with the critical support of (allogeneic) MSC and secreted factors.^64^ Nomenclature was officially changed and consolidated by the International Society for Cell and Gene Therapy (ISCT) in 2019,^65^ 15 years after a panel of experts delineated the release criteria to identify MSC in 2006.^66^ Mesenchymal stromal cells were defined as “mesenchymal” elements since embedded into a large extracellular compartment. Mesenchymal cells are characterized by few intercellular adhesion and lack of polarity. Furthermore, MSCs have been called “stromal” cells since they can actively play a role in producing new stroma and, as fibroblastic cells, are capable of adhering to plastic. Morphologically, MSCs are described as long and thin cells with a large nucleus that is surrounded by finely divided chromatin particles, giving the nucleus a clear appearance. Perinatal MSCs have also been identified “according to the criteria of The Mesenchymal and Tissue Stem Cell Committee of the International Cell Therapy Association.” The widely accepted minimal criteria to define MSC was proposed by ISCT experts^66^ in 2006. Such minimal criteria comprise (1) plastic-adhesion in vitro; (2) characteristic expression of surface markers such as ectoenzymes and receptors CD73, CD90, and CD105; (3) ability to mature into mesoderm lineages such as adipocytes, osteoblasts, and chondroblasts. However, these multipotent cells do not mature into the hematopoietic lineage as well as several other lineages. But general consensus or final proof in such regard is still pending.

The topic where consensus has been reached and no more discussion has been raised concerns MSC as an ATMP product. Despite the vast interest in perinatal MSC, there is still a lack of standardized protocols and guidelines for isolation, preservation, expansion, and delivery to ensure the large-scale production of cells for clinical uses.^67^ Similar considerations have been offered for any other cell products potentially releasable from perinatal tissues, such as hAEC or hUVEC. The processing of all the PnD cells must be performed in a controlled environment with filtered laminar flow and in compliance with GMP and GLP rules to ensure maximum sterility of the working space.^68^ During the last years, several groups performed a critical review of the manufacturing process, aimed to standardize reagents and SOPs in accordance with current GMP requirements.^12,69^ Additional refinements and studies focused on the expansion of the pool of primary tissue by donor tissue hypothermic preservation (to extend the time allowed between collection and processing), as well as optimization and harmonization of cryogenic preservation of cells and tissues for clinical use (DMSO-free solutions have been proposed and under investigation). It is important to note, that PnD populations may vary depending on the donor, so isolation and qualification protocols might require further optimization. Regarding PnD tissue specimens, mechanical dissection, and surgical resections are commonly preparing different tissues and specimens to further enzymatic isolation, and eventually to gradient purification. All the perinatal stem/progenitor cells undergo the same validation criteria as any other ATMP: sterility test; cell viability quantification; and evaluation of cell suspension identity to determine the grade of heterogeneity within the final product.

The route of infusion and more relevantly the transplantation of PnD generated from different donors in one single recipient has also been tested and largely debated. According to European recommended guidelines (EDQM-Guide), the pooling of allogeneic preparations should be limited or avoided, when possible. However, in order to reach the cell dose required to generate clinical effects, in the past, injections of multiple donors have been offered and described. In such exceptions, the final (cellular or tissue) product was offered to grant clinical outcomes. Obviously, in such combined therapies, a comprehensive risk-benefit analysis must be performed to determine whether this procedure is justified, and risks mitigated. The sequential transplantation of different donors, rather than a pool of allogenic cell products, has been described as beneficial in cord blood transfusion and it is currently a common and recommended routine. Similarly, in other allogenic cell therapies (eg, hepatocyte transplantation), the sequential injection of different donors, in some cases more than 10 donors, has been reported effective and safe. However, the co-infusion or repeated injection of products generated by allogeneic donors is commonly seen as risky and should be properly evaluated. Furthermore, such multi-product use must be coupled with complete traceability of all the individual donations.

## Conclusion

As for any new medicinal product, the development and approval of clinical batches need to be based on non-clinical data supportive of the mechanism of action and potential therapeutic activity. Many PnD products, including multipotent MSC or hAEC, have complex and not fully characterized MoA, making it difficult to determine which product attributes are most relevant to clinical purposes. Positive clinical results could be the outcome of one or more different mechanisms of action. Appropriate laboratory analyses testing the biological activities should be based on the intended biological effect, and on clinical response. Since several different diseases are commonly treated using PnD, there is no single test that can adequately measure product attributes to predict clinical efficacy. No recommendations are present in any guidelines, since International Agencies recognize the actual limitations and clearly state that a single biological or analytical assay may not be sufficient to provide an adequate measure of potency.^62^ Several reports have highlighted such limitations and offered novel strategies to overcome such a burden. During the past years, within COST action, PnD experts within all the European Countries gathered together and shared their experiences and protocols.^62^ It has been reported as a battery of assays, rather than a singular analysis, which is preferable and more informative to evaluate specific functions requested to correct the disease and to better match recipients’ needs. A multi-disciplinary approach should be implemented and optimized to ensure a complete and useful PnD profile: quantitative transcriptomic analysis; and flow cytometric analysis of functionally relevant surface markers. When pertinent, protein-based assay of secretome and cytoplasmatic enzymes.

## Supplementary Material

szad068_suppl_Supplementary_Figure_1

## Data Availability

No new data were generated or analyzed in support of this research.

## References

[1] Silini AR , Di Pietro R, Lang-Olip I, et al. Perinatal derivatives: where do we stand? A roadmap of the human placenta and consensus for tissue and cell nomenclature. Front Bioeng Biotechnol. 2020;8:610544. 10.3389/fbioe.2020.610544 33392174 PMC7773933

[2] Davis J. Skin transplantation with a review of 550 cases at the John Hopkins Hospital. John Hopkins Med J. 1910;15(1):307.

[3] Gluckman E , Broxmeyer HA, Auerbach AD, et al. Hematopoietic reconstitution in a patient with Fanconi’s anemia by means of umbilical-cord blood from an HLA-identical sibling. N Engl J Med. 1989;321(17):1174-1178. 10.1056/NEJM198910263211707 2571931

[4] https://www.sprint-cost.org/

[5] Stroncek DF , England L. Protecting the health and safety of cell and tissue donors. ISBT Sci Ser. 2015;10(Suppl 1):108-114. 10.1111/voxs.12150 25937830 PMC4414045

[6] O’Donnell PV , Pedersen TL, Confer DL, et al; Donor Health and Safety Working Committee from Center for International Blood and Marrow Transplant Research (CIBMTR). Practice patterns for evaluation, consent, and care of related donors and recipients at hematopoietic cell transplantation centers in the United States. Blood. 2010;115(24):5097-5101. 10.1182/blood-2010-01-262915 20228276 PMC2890146

[7] Commission Directive 2006/17/EC of 8 February 2006 implementing Directive 2004/23/EC of the European Parliament and of the Council as regards certain technical requirements for the donation, procurement and testing of human tissues and cells (Text with EEA relevance). (OJ L 38 28.11.2006, p. 40, ELI: http://data.europa.eu/eli/dir/2006/17/oj)

[8] European Union 2004 Directive 2004/23/EC of the European Parliament and of the Council of 31 March 2004 on setting standards of quality and safety for the donation, procurement, testing, processing, preservation, storage, and distribution of human tissues and cells. Official Journal of the European Union L102/48.

[9] European Union 2002 Directive 2002/98/EC of the European Parliament and of the Council of 27 January 2003 on setting standards of quality and safety for the collection, testing, processing, storage and distribution of human blood and blood components and amending Directive 2001/83/EC. Official Journal of the European Union L33/30.

[10] EuroGTP Project. available at www.goodtissuepractices.eu. Accessed April 13, 2022

[11] Tyszkiewicz J , Uhrynowska-Tyszkiewicz I, Kaminski A, Dziedzic-Goclawska A. Amnion allografts prepared in the Central Tissue Bank in Warsaw. Ann Transplant. 1999;4(3-4):85-90. 10853790

[12] Gramignoli R , Srinivasan RC, Kannisto K, Strom SC. Isolation of human amnion epithelial cells according to current good manufacturing procedures. Curr Protoc Stem Cell Biol. 2016 12;37:1E.10.1-1E.10.13. 10.1002/cpsc.2 27171794

[13] Eudralex [collection of rules and regulations governing medicinal products in the European Union]. Volume 4, EU guidelines on good manufacturing practice specific to advanced therapy medicinal products. available at http://ec.europa.eu/health/sites/health/files/files/eudralex/vol-4/2017_11_22_guidelines_gmp_for_atmps.pdf. Accessed May 15, 2019.

[14] Zanini C , Severina F, Lando G, et al. Good design practices for an integrated containment and production system for advanced therapies. Biotechnol Bioeng. 2020;117(8):2319-2330. 10.1002/bit.27376 32374459

[15] Gindraux F , Hofmann N, Agudo-Barriuso M, et al. Perinatal derivatives application: identifying possibilities for clinical use. Front Bioeng Biotechnol. 2022;10:977590. 10.3389/fbioe.2022.977590 36304904 PMC9595339

[16] EMA/CAT/285241/2010 rev.1. Committee for Advanced Therapies (CAT). Reflection paper on classification of advanced therapy medicinal products.

[17] Yang H , Li N, Chang S. A risk-based approach to setting sterile filtration bioburden limits. PDA J Pharm Sci Technol. 2013;67(6):601-609. 10.5731/pdajpst.2013.00942 24265301

[18] Regulation EC1394/2007 of the European Parliament and of the Council of 13 November 2007 on Advanced Therapy Medicinal Products and Amending Directive 2001/83/EC and Regulation (EC) No 726/2004. 2007.

[19] EMA/CHMP/ICH/24235/2006. ICH guideline Q9 on quality risk management.

[20] Klop AC , Vester MEM, Colman KL, et al. The effect of repeated freeze-thaw cycles on human muscle tissue visualized by postmortem computed tomography (PMCT). Clin Anat. 2017;30(6):799-804. 10.1002/ca.22917 28514529

[21] Lee SH , Tseng SC. Amniotic membrane transplantation for persistent epithelial defects with ulceration. Am J Ophthalmol. 1997;123(3):303-312. 10.1016/s0002-9394(14)70125-4 9063239

[22] Shimazaki J , Shinozaki N, Tsubota K. Transplantation of amniotic membrane and limbal autograft for patients with recurrent pterygium associated with symblepharon. Br J Ophthalmol. 1998;82(3):235-240. 10.1136/bjo.82.3.235 9602618 PMC1722509

[23] Hennerbichler S , Reichl B, Pleiner D, et al. The influence of various storage conditions on cell viability in amniotic membrane. Cell Tissue Bank. 2007;8(1):1-8. 10.1007/s10561-006-9002-3 16807768

[24] Pogozhykh O , Hofmann N, Gryshkov O, et al. Repeated freezing procedures preserve structural and functional properties of amniotic membrane for application in ophthalmology. Int J Mol Sci. 2020;21(11):4029. 10.3390/ijms21114029 32512889 PMC7312941

[25] Von Versen-Höynck F , Hesselbarth U, Möller DE. Application of sterilised human amnion for reconstruction of the ocular surface. Cell Tissue Bank. 2004;5(1):57-65. 10.1023/b:catb.0000022222.41304.de 15256840

[26] Singh R , Gupta P, Kumar P, Kumar A, Chacharkar MP. Properties of air dried radiation processed amniotic membranes under different storage conditions. Cell Tissue Bank. 2003;4(2-4):95-100. 10.1023/B:CATB.0000007030.72031.12 15256845

[27] Singh R , Singh D, Singh A. Radiation sterilization of tissue allografts: a review. World J Radiol. 2016 Apr 28;8(4):355-369. 10.4329/wjr.v8.i4.355 27158422 PMC4840193

[28] Mao Y , Hoffman T, Dhall S, et al. Endogenous viable cells in lyopreserved amnion retain differentiation potential and anti-fibrotic activity in vitro. Acta Biomater. 2019;94:330-339. 10.1016/j.actbio.2019.06.002. Epub 2019 Jun 6 31176843

[29] Ross A , Kearney JN. The measurement of water activity in allogeneic skin grafts preserved using high concentration glycerol or propylene glycol. Cell Tissue Bank. 2004;5(1):37-44. 10.1023/b:catb.0000022284.53499.59 15256838

[30] Huang Q , Pegg DE, Kearney JN. Banking of non-viable skin allografts using high concentration of glycerol or propylene glycol. Cell Tissue Bank. 2004;5(1):3-21. 10.1023/b:catb.0000022234.02322.13 15256836

[31] Rejzek A , Weyer F, Eichberger R, Gebhart W. Physical changes of amniotic membranes through glycerolization for the use as an epidermal substitute Light and electron microscopic studies. Cell Tissue Bank. 2001;2(2):95-102. 10.1023/A:1014316232009 15256920

[32] Ginis I , Grinblat B, Shirvan MH. Evaluation of bone marrow-derived mesenchymal stem cells after cryopreservation and hypothermic storage in clinically safe medium . Tissue Eng Part C Methods. 2012;18(6):453-463. 10.1089/ten.TEC.2011.0395 22196031

[33] Corwin WL , Baust JM, Baust JG, Van Buskirk RG. Characterization and modulation of human mesenchymal stem cell stress pathway response following hypothermic storage. Cryobiology. 2014;68(2):215-226. 10.1016/j.cryobiol.2014.01.014 24508650 PMC4001798

[34] Linkova DD , Rubtsova YP, Egorikhina MN. Cryostorage of mesenchymal stem cells and biomedical cell-based products. Cells. 2022;11(17):2691. 10.3390/cells11172691 36078098 PMC9454587

[35] Mutsenko V , Barlič A, Pezić T, et al. Me2SO- and serum-free cryopreservation of human umbilical cord mesenchymal stem cells using electroporation-assisted delivery of sugars. Cryobiology. 2019;91:104-114. 10.1016/j.cryobiol.2019.10.002 31593692

[36] Wang J , Zhao G, Zhang Z, Xu X, He X. Magnetic induction heating of superparamagnetic nanoparticles during rewarming augments the recovery of hUCM-MSCs cryopreserved by vitrification. Acta Biomater. 2016;33:264-274. 10.1016/j.actbio.2016.01.026 26802443 PMC5500173

[37] Srinivasan RC , Strom SC, Gramignoli R. Effects of cryogenic storage on human amnion epithelial cells. Cells. 2020;9(7):1696. 10.3390/cells9071696 32679793 PMC7407665

[38] Lauterboeck L , Wolkers WF, Glasmacher B. Cryobiological parameters of multipotent stromal cells obtained from different sources. Cryobiology. 2017;74:93-102. 10.1016/j.cryobiol.2016.11.009 27916562

[39] Moll G , Geißler S, Catar R, et al. Cryopreserved or fresh mesenchymal stromal cells: only a matter of taste or key to unleash the full clinical potential of MSC therapy? Adv Exp Med Biol. 2016;951:77-98. 10.1007/978-3-319-45457-3_7 27837556

[40] François M , Copland IB, Yuan S, et al. Cryopreserved mesenchymal stromal cells display impaired immunosuppressive properties as a result of heat-shock response and impaired interferon-γ licensing. Cytotherapy. 2012;14(2):147-152. 10.3109/14653249.2011.623691 22029655 PMC3279133

[41] Oja S , Kaartinen T, Ahti M, et al. The utilization of freezing steps in mesenchymal stromal cell (MSC) manufacturing: potential impact on quality and cell functionality attributes. Front Immunol. 2019;10:1627. 10.3389/fimmu.2019.01627 31379832 PMC6646664

[42] Pollock K , Sumstad D, Kadidlo D, McKenna DH, Hubel A. Clinical mesenchymal stromal cell products undergo functional changes in response to freezing. Cytotherapy. 2015;17(1):38-45. 10.1016/j.jcyt.2014.06.008 25457275 PMC4274232

[43] Chinnadurai R , Copland IB, Garcia MA, et al. Cryopreserved mesenchymal stromal cells are susceptible to T-cell mediated apoptosis which is partly rescued by IFNγ licensing. Stem Cells. 2016;34(9):2429-2442. 10.1002/stem.2415 27299362 PMC5016228

[44] Semenova E , Grudniak MP, Bocian K, et al. Banking of AT-MSC and its influence on their application to clinical procedures. Front Bioeng Biotechnol. 2021;9(9):773123. 10.3389/fbioe.2021.773123 34917599 PMC8670380

[45] Marquez-Curtis LA , Janowska-Wieczorek A, McGann LE, Elliott JA. Mesenchymal stromal cells derived from various tissues: biological, clinical and cryopreservation aspects. Cryobiology. 2015;71(2):181-197. 10.1016/j.cryobiol.2015.07.003 26186998

[46] Cottle C , Porter AP, Lipat A, et al. Impact of cryopreservation and freeze-thawing on therapeutic properties of mesenchymal stromal/stem cells and other common cellular therapeutics. Curr Stem Cell Rep. 2022;8(2):72-92. 10.1007/s40778-022-00212-1 35502223 PMC9045030

[47] Srinivasan RC , Kannisto K, Strom SC, Gramignoli R. Evaluation of different routes of administration and bio-distribution of human amnion epithelial cells in mice. Cytotherapy. 2019;21(1):113-124. 10.1016/j.jcyt.2018.10.007 30409699

[48] Galipeau J. The mesenchymal stromal cells dilemma--does a negative phase III trial of random donor mesenchymal stromal cells in steroid-resistant graft-versus-host disease represent a death knell or a bump in the road? Cytotherapy. 2013 Jan;15(1):2-8. 10.1016/j.jcyt.2012.10.002 23260081

[49] Puzanov MV , Vasilyeva LB, Popova PV, Grineva EN, Dmitrieva RI. New approach to cryopreservation of primary noncultivated human umbilical vein endothelium in biobanking. Biopreserv Biobank. 2018;16(2):114-119. 10.1089/bio.2017.0086 29363992

[50] Théry C , Ostrowski M, Segura E. Membrane vesicles as conveyors of immune responses. Nat Rev Immunol. 2009;9(8):581-593. 10.1038/nri2567 19498381

[51] Brennan M , Layrolle P, Mooney DJ. “Biomaterials functionalized with MSC secreted extracellular vesicles and soluble factors for tissue regeneration”. Adv Funct Mater. 2020;30(37):1909125. 10.1002/adfm.201909125 32952493 PMC7494127

[52] Aharon A , Tamari T, Brenner B. “Monocyte-derived microparticles and exosomes induce procoagulant and apoptotic effects on endothelial cells”. Thromb Haemost. 2008;100(5):878-885. 10.1160/th07-11-0691 18989533

[53] Del Conde I , Shrimpton CN, Thiagarajan P, López JA. Tissue-factor-bearing microvesicles arise from lipid rafts and fuse with activated platelets to initiate coagulation. Blood. 20051;106(5):1604-1611. 10.1182/blood-2004-03-1095 15741221

[54] Principles of Microbiological Testing, Chapter 11. Guide to the Quality and Safety of Tissues and Cells for Human Application, 5th edition, Strasbourg, France. European Directorate for the Quality of Medicines and HealthCare; Council of Europe, 2022.

[55] Sterility, General Chapter, 2.6.1 Ph. Eur., 10th edition. Strasbourg, France: Council of Europe 2020.

[56] Guidelines for Using the Test for Sterility, General Chapter, 5.1.9 Ph. Eur., 10th edition. Strasbourg, France: Council of Europe 2020.

[57] Microbiological Examination of Non-Sterile Products (Total Viable Aerobic Count), General Chapter 2.6.12 Ph. Eur., 10th edition. Strasbourg, France: Council of Europe 2020.

[58] Microbiological Examination of Non-Sterile Products (Test for Specified Micro-Organisms), General Chapter 2.6.13. Ph. Eur., 10th edition. Strasbourg, France: Council of Europe 2020.

[59] Mycoplasmas, General Chapter. 2.6.7 Ph. Eur., 10th edition. Strasbourg, France: Council of Europe 2020.

[60] Bacterial Endotoxins, General Chapter. 2.6.14 Ph. Eur., 10th edition. Strasbourg, France: Council of Europe 2020.

[61] Guidelines for Using the Test for Bacterial Endotoxins, General Chapter. 5.1.10 Ph. Eur., 10th edition. Strasbourg, France: Council of Europe 2020.

[62] Pozzobon M , D’Agostino S, Roubelakis MG, et al. General consensus on multimodal functions and validation analysis of perinatal derivatives for regenerative medicine applications. Front Bioeng Biotechnol. 2022;10:961987. 10.3389/fbioe.2022.961987 36263355 PMC9574482

[63] Caplan AI. Mesenchymal stem cells. J Orthop Res. 1991;9(5):641-650. 10.1002/jor.1100090504 1870029

[64] Caplan AI. Mesenchymal stem cells: time to change the name! Stem Cells Transl Med. 2017;6(6):1445-1451. 10.1002/sctm.17-0051 28452204 PMC5689741

[65] Viswanathan S , Shi Y, Galipeau J, et al. Mesenchymal stem versus stromal cells: International Society for Cell & Gene Therapy (ISCT®) Mesenchymal Stromal Cell committee position statement on nomenclature. Cytotherapy. 2019;21(10):1019-1024. 10.1016/j.jcyt.2019.08.002 31526643

[66] Dominici M , Le Blanc K, Mueller I, et al. Minimal criteria for defining multipotent mesenchymal stromal cells The International Society for Cellular Therapy position statement. Cytotherapy. 2006;8(4):315-317. 10.1080/14653240600855905 16923606

[67] Wang Y , Han ZB, Song YP, Han ZC. Safety of mesenchymal stem cells for clinical application. Stem Cells Int. 2012;2012:652034. 22685475 10.1155/2012/652034 PMC3363282

[68] Krampera M , Franchini M, Pizzolo G, Aprili G. Mesenchymal stem cells: from biology to clinical use. Blood Transfus. 2007;5(3):120-129. 10.2450/2007.0029-07 19204764 PMC2535891

[69] Murphy S , Rosli S, Acharya R, et al. Amnion epithelial cell isolation and characterization for clinical use. Curr Protoc Stem Cell Biol. 2010;Chapter 1:Unit 1E.6. 10.1002/9780470151808.sc01e06s13 20373516

