# Supplementary figures and images for: Expert Revision of Key Elements for Clinical-Grade Production and Qualification of Perinatal Derivatives
Source: Stem Cells Transl Med. 2023 Dec 10;13(1):14–29. doi: 10.1093/stcltm/szad068 (PMC10785218; doi:10.1093/stcltm/szad068)

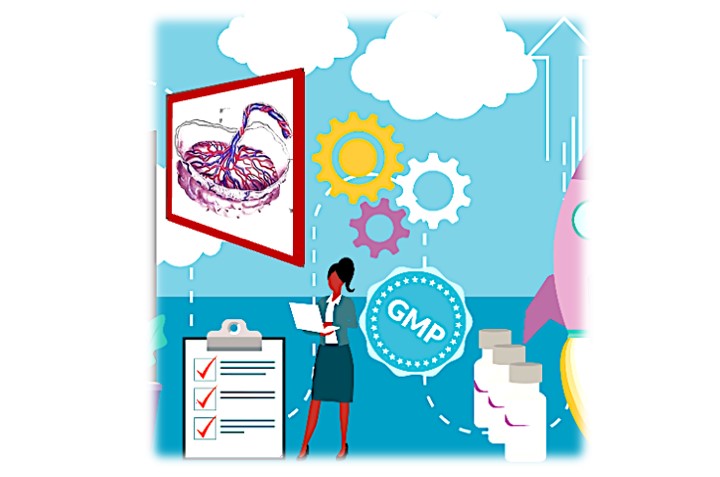

Supplement: szad068_suppl_Supplementary_Figure_1 [file szad068_suppl_supplementary_figure_1.jpeg]
